# Venom Proteome of Spine-Bellied Sea Snake (*Hydrophis curtus*) from Penang, Malaysia: Toxicity Correlation, Immunoprofiling and Cross-Neutralization by Sea Snake Antivenom

**DOI:** 10.3390/toxins11010003

**Published:** 2018-12-23

**Authors:** Choo Hock Tan, Kae Yi Tan, Tzu Shan Ng, Si Mui Sim, Nget Hong Tan

**Affiliations:** 1 Department of Pharmacology, Faculty of Medicine, University of Malaya, Kuala Lumpur 50603, Malaysia; debrasim@um.edu.my; 2 Department of Molecular Medicine, Faculty of Medicine, University of Malaya, Kuala Lumpur 50603, Malaysia; kytan_kae@um.edu.my (K.Y.T.); ngtzushan@um.edu.my (T.S.N.); tanngethong@yahoo.com.sg (N.H.T.)

**Keywords:** *Lapemis hardwickii*, immunoreactivity, alpha-neurotoxins, three-finger toxins, phospholipase A_2_, envenomation, neutralization

## Abstract

The venom proteome of *Hydrophis curtus* (synonym: *Lapemis hardwickii*) from Penang, Malaysia was investigated with nano-electrospray ionization-liquid chromatography tandem mass spectrometry (ESI-LCMS/MS) of the reverse-phase high-performance liquid chromatography (HPLC) venom fractions. Thirty distinct protein forms were identified as toxins from ten families. The three major protein families were phospholipase A_2_ (PLA_2_, 62.0% of total venom proteins), three-finger toxin (3FTX, 26.33%) and cysteine-rich secretory protein (CRiSP, 9.00%). PLA_2_ comprises diverse homologues (11 forms), predominantly the acidic subtypes (48.26%). 3FTX composed of one short alpha-neurotoxin (SNTX, 22.89%) and four long alpha-neurotoxins (LNTX, 3.44%). Both SNTX and LNTX were lethal in mice (intravenous LD_50_ = 0.10 and 0.24 μg/g, respectively) but the PLA_2_ were non-lethal (LD_50_ >1 μg/g). The more abundant and toxic SNTX appeared to be the main driver of venom lethality (holovenom LD_50_ = 0.20 μg/g). The heterologous Sea Snake Antivenom (SSAV, Australia) effectively cross-neutralized the venom (normalized potency = 9.35 mg venom neutralized per g antivenom) and the two neurotoxins in vivo, with the LNTX being neutralized more effectively (normalized potency = 3.5 mg toxin/g antivenom) than SNTX (normalized potency = 1.57 mg/g). SSAV immunorecognition was strong toward PLA_2_ but moderate-to-weak toward the alpha-neurotoxins, indicating that neutralization of the alpha-neurotoxins should be further improved.

## 1. Introduction

The Elapidae family of venomous snakes consists of approximately 369 species in >60 genera (www.reptile-database.org). A basal split in their phylogeny circa 10.1–24.3 Mya gave rise to the recent divergence of Australo-Melanesian elapids, resulting in two distinct subfamilies: (1) Elapinae, consisting of the paleogeographically-related Asian, African and American elapids; (2) Hydrophiinae, comprising the Australo-Melanesian elapids [1,2]. The Hydrophiinae represents a diverse paraphyletic clade that rapidly radiates in and around Australia, producing lineages that were well adapted to terrestrial or aquatic habitats [2]. The aquatic elapids, generally referred to as sea snakes, form a large group of marine reptiles. It comprises the basal, semi-aquatic sea kraits (*Laticauda* sp., 6 species) and the more derived, densely complex true sea snakes (>50 species). The genus *Hydrophis* constitutes the core group of the true sea snakes. It is considered as a monophyletic clade by consensus today [3].

*Hydrophis* sea snakes live their entire lives in the water, and some of the species are often part of the by-catch of fishermen in areas where they occur. Sea snake bite typically occurs among the fishermen while removing sea snakes entangled in their fishing nets—a classic scenario described in most sea snake bite literature [4,5], although treading on a sea snake in shallow estuaries has also been reported as a cause [6,7]. In Southeast Asia, sea snake envenoming is increasingly an occupational health hazard that has expanded its threat beyond the fishing community. In this part of the world, sea snakes are a significant part of the global wildlife trade [8], where they are captured, bred and harvested for live snake parts used in the production of accessories, and for food (sea snake meat as an exotic delicacy), as well as for medicinal use (tonic soup, snake wine, gall bladder as traditional medicament). The increased human contact with sea snakes from these anthropogenic activities poses a threat to a wider community of people. Although sea snake bites are infrequently reported, envenoming is fatal, and early treatment may be missed as the bite is painless [9]. Clinically, patients envenomed by the common beaked sea snake (*Hydrophis schistosus*, or *Enhydrina schistosa*) develop neuromuscular paralysis and systemic myotoxicity complicated by acute kidney injury [10,11]. The pathophysiology of *H. schistosus* envenoming has been correlated with the presence of alpha-neurotoxins and basic phospholipases A_2_ in the venom [12,13,14], while the envenomation by other sea snake species is commonly assumed to follow a similar mechanism. As sea snakes are highly adapted to their ecological niche, their venoms are thought to be “biochemically simple”, with a composition that is strictly diet-driven to subdue fast-moving prey (fish). The streamlined nature of sea snake venoms and the link to their ichthyophagous dietary simplicity have been shown earlier in an extensive liquid chromatography-mass spectrometry (LC/MS) analysis of Colubroidea snake venoms [15]. Indeed, a handful of venom proteomic studies of sea snakes and a sea krait were in agreement that alpha-neurotoxins and phospholipases A_2_ form the dominant proteins in these venoms [12,16,17,18,19,20]. The highly streamlined “minimalist” venom proteomes, however, does not limit the diversity of the toxins: the molecular subtypes, relative abundances and functionality (including toxic activities) of the venom proteins could still vary [14,19,21,22]. This provides the rationale for continuous investigation into the venom proteomes of different sea snake species in order to understand the intra-genus and intra-species diversity and evolution of the toxins. The knowledge will pave the path for in-depth exploration of venom antigenic properties, which is crucial for the optimization of antivenom production technique in the region [23].

Currently, the only definitive antidote treatment for sea snake envenoming is the Sea Snake Antivenom (SSAV) produced by Seqirus (previously CSL, Australia) against *Hydrophis schistosus*, using venom sourced from Penang Island, Malaysia. This antivenom has been shown to be effective in vitro in preventing neuromuscular depressant activity induced by the venoms of several sea snakes and a sea krait [24]. In vivo, SSAV was able to protect mice from the lethal effect of *H. schistosus*, *Hydrophis platura*, *Aipysurus laevus* and *Laticauda colubrina* venoms to various extents [12,17,18,19]. To elucidate the limiting factors of antivenom efficacy, the principal toxins, namely the subtypes of alpha-neurotoxins and phospholipases A_2_ in *H. schistosus* and *L. colubrina* venoms, have been further purified and tested for toxin-specific neutralization [19,25]. The in vivo cross-neutralization of SSAV against the venoms and principal toxins of other sea snake species that are medically relevant and widely distributed, e.g., the spine-bellied sea snake (*Hydrophis curtus*), remains to be further examined.

**Figure 1:**
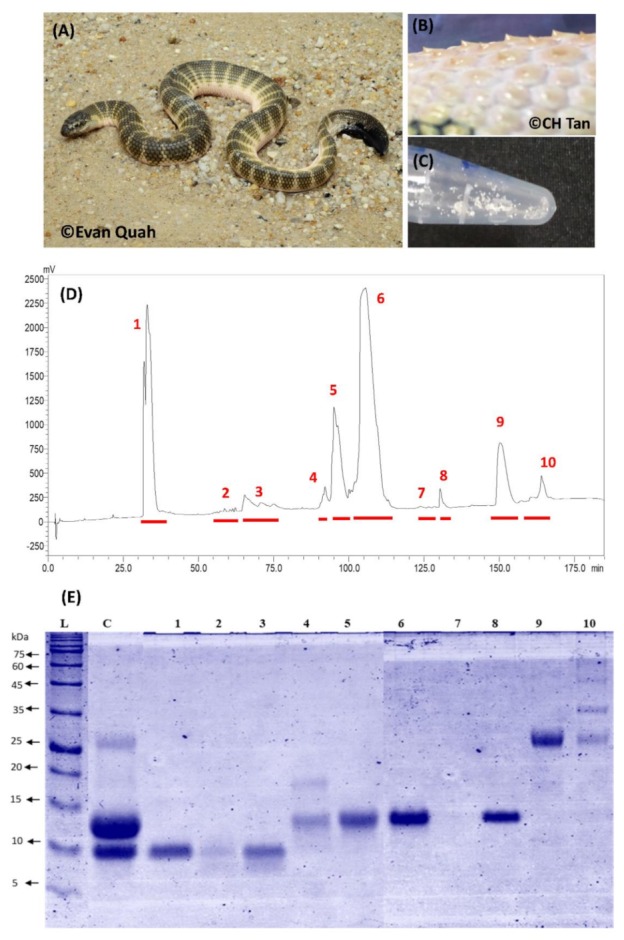
(**A**) Spine-bellied sea snake, *Hydrophis curtus* from Penang Island, Malaysia. (**B**) Protruding spine-like structures on the ventral scales of the snake. (**C**) Lyophilized *H. curtus* venom in whitish crystalline form. (**D**) Decomplexation of *H. curtus* (Penang, Malaysia) venom with C18 reverse-phase HPLC. (**E**) 15% SDS-PAGE of *H. curtus* venom and its HPLC fractions under reducing conditions. L: Protein markers; C: Whole venom. Numbers indicate HPLC fractions.

In the Gulf of Thailand, it was reported that one of the main species involved in the catch and trade industry is the spine-bellied sea snake [8]—named for the multiple small spiny protrusion on its ventral scales over the abdominal region (Figure 1). This species was previously placed within the genus *Lapemis*, which is now incorporated into the core *Hydrophis* group, and is known as *H. curtus* (Shaw’s sea snake) or *Hydrophis hardwickii* (Hardwick’s sea snake), although *H. hardwickii* is commonly considered to be a synonym of *H. curtus* or a subspecies of *H. curtus*. *Hydrophis curtus* has extensive distribution form the Persian Gulf to the Indian coastline, Myanmar, Thailand, Straits of Malacca, Strait of Taiwan, South China Sea, the Philippines, Indonesia, Papua New Guinea, and northern and eastern Australia (http://reptile-database.reptarium.cz/). This is also a species commonly encountered by fishermen in the western coast of Peninsular Malaya [26], as with the beaked sea snake, *H. schistosus* (author CHT’s observation). Recently, the venom proteome of *H. curtus* from Australian waters (Weipa) has been reported. It shows a venom profile rich in alpha-neurotoxins, phospholipases A_2_ and cysteine-rich venom proteins, along with some high molecular weight proteins and a number of regulatory or cellular proteins [20]. In the present study, using a decomplexing proteomic approach, the venom proteome of *H. curtus* from Penang (Malaysia) was characterized for insights into the geographical variation of the venom. The in vivo toxicity and the antigenic properties of the major toxins of this species were also investigated, and the venom functionality was elucidated in correlation with the venom proteome. In addition, the in vivo efficacy of the heterologous antivenom, SSAV, was also examined for cross-neutralization and immunorecognition for *H. curtus* venom proteins, including the principal lethal toxins.

## 2. Results

### 2.1. Decomplexation Proteomics of Penang H. curtus Venom

*H. curtus* venom was resolved by C18 reverse-phase high-performance liquid chromatography (HPLC) into 10 protein fractions as assigned in Figure 1. Fractions 1, 5, 6 and 9 were the major fractions, constituting >90% total venom proteins (estimation made by peaks area). SDS-PAGE showed that fractions 1–3 contained proteins of low molecular mass (<10 kDa), whereas Fractions 4, 5, 6 and 8 contained proteins of 12–15 kDa (Figure 1). Moderate to high molecular weight proteins eluted later (beyond 150 min of HPLC): Fraction 9 contained a protein of approximately 26 kDa, while Fraction 10 was heterogeneous with multiple proteins (25–100 kDa). There was no protein band observed in Fraction 7. The SDS-PAGE of the whole venom revealed that the proteins of 12–15 kDa formed the bulk of the venom proteins (~60% by densitometry), followed by proteins of <10 kDa (~30%) and 26 kDa (~10%), consistent with the HPLC profile and the overall SDS-PAGE of the venom fractions (Figure 1).

### 2.2. Proteome of Penang H. curtus Venom

The HPLC fractions of venom proteins were further identified by LCMS/MS and data-mining through a non-redundant NCBI protein dataset (taxonomy: Serpentes, taxid: 8570) and an in-house venom-gland transcriptomic database. Table 1 documents the identified and categorized proteins according to the corresponding HPLC fractions. In the table, relevant information on the protein profiling, including the protein abundances (% total venom proteins), accession numbers, protein scores and numbers of matched tryptic peptides is shown. Data generated via the tandem mass spectrometry (MS/MS) e.g., the ionic mass/charge ratios and tryptic peptide sequences, were compiled in Supplementary File Table S1.

**Figure 2:**
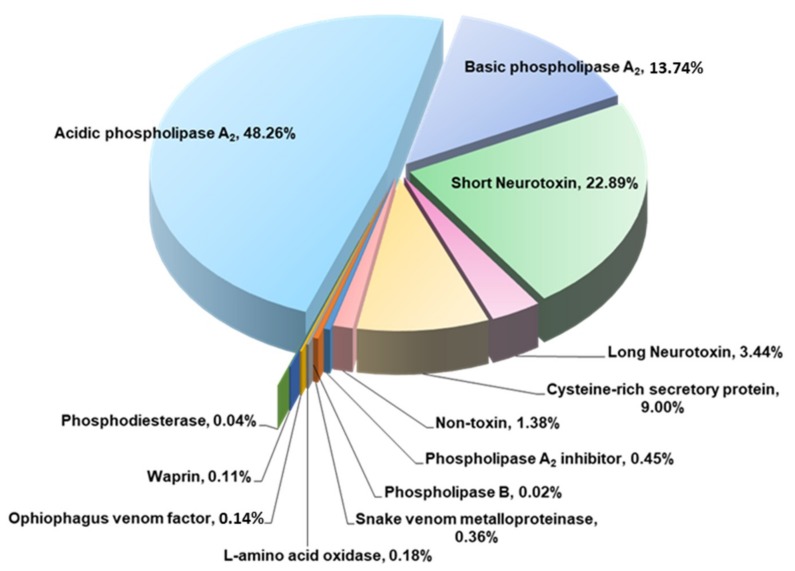
Venom proteome of the spine-bellied sea snake, *Hydrophis curtus* from Penang Island, Malaysia. Percentages indicate relative protein abundance by total venom proteins.

A total of 41 distinct proteins (toxins and non-toxins) were identified from *H. curtus* venom (Table 2). The majority of the proteins identified (26 out of 41) were annotated to database sequences shared by the same species (*H. curtus* or *H. hardwickii*). In total, 98.6% of the venom proteins were composed of toxins clustered into 10 families (comprising 30 identifiable toxins). Of these, phospholipase A_2_ (PLA_2_, 62.0%) dominated the proteome. Within the PLA_2_ family, the acidic subtypes were more abundant than the basic subtypes (5:1 ratio). The protein abundance was followed by three-finger toxin (3FTX, 26.33%) and cysteine-rich secretory protein (CRiSP, 9.0%). The 3FTX proteins consisted of short neurotoxin (SNTX, one subtype) as its major component (22.89%), while the long neurotoxins (LNTX, 3 subtypes) were less abundant (3.44%). A variety of toxins of low abundances (<0.5% each) were also identified, including snake venom metalloproteinase, L-amino acid oxidase, Ophiophagus venom factor, waprin, phosphodiesterase, phospholipase A_2_ inhibitor and phospholipase B (Figure 2). Approximately 11 non-toxin proteins or 1.4% of the venom proteins were regulatory or cellular proteins.

The proteomic profile of the Malaysian *H. curtus* venom (current study) was further tabulated in Table 3 in comparison with venomic data reported previously for other sea elapids.

### 2.3. Immunoreactivity Profiling of H. curtus Venom Proteins

**Figure 3:**
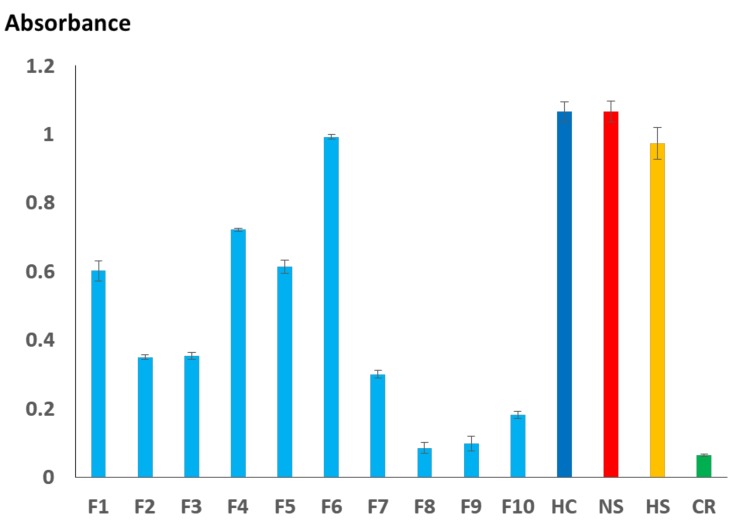
Immunoprofiling of *H. curtus* venom and protein fractions by the heterologous Sea Snake Antivenom. F1–F10: Reverse-phase HPLC of *H. curtus* venom; HC: *H. curtus* venom; NS: *Notechis scutatus* venom; HS: *Hydrophis schistosus* venom; CR: *Calloselasma rhodostoma* venom.

Figure 3 shows the immunoreactivity of SSAV toward the different venom fractions (10 ng protein) of *H. curtus*, the holovenoms of *H. curtus*, *Notechis scutatus*, *H. schistosus* and *C. rhodostoma*. The binding activity of SSAV (reflected by the absorbance on ELISA) toward *H. curtus* and *N. scutatus* venoms were high (Abs >1.0) but marginally lower toward *H. schistosus* venom. The negative control, *C. rhodotoma* venom, exhibited weak binding by SSAV (Abs ~0.1). Among the protein fractions, Fraction 6 (containing the major PLA_2_ proteins) immunoreacted most strongly with SSAV (Abs ~1.0). Fractions 1 (SNTX), 4 and 5 (PLA2) showed moderate immunoreactivity (Abs: 0.6–0.7), whereas Fractions 2, 3 (LNTX) and 7 (non-toxin components) were relatively weak in their immunoreactivity (Abs ~0.3). SSAV binding activity toward proteins in Fractions 8 (non-toxin), 9 (CRISP) and 10 (enzymes, inhibitors and non-toxin components) were considerably low, with average absorbance values of <0.2.

### 2.4. Toxicity of H. curtus Venom and Principal Toxins

The Malaysian *H. curtus* venom was lethal in mice with an intravenous LD_50_ of 0.20 (0.18–0.24) µg/g. The SNTX (F1) and LNTX (F3) were both lethal, and the SNTX appeared to be more toxic (LD_50_ = 0.10 µg/g) than LNTX (LD_50_ = 0.24 µg/g). Screening of toxicity for all other fractions including the major fractions F4 and F6 which contained primarily PLA_2_, as well as F9 which contained CRISP, showed that these proteins were non-lethal in mice at a dose >1 µg/g.

### 2.5. Cross-Neutralization of H. curtus Venom and Toxins by SSAV

In neutralization assay, SSAV cross-neutralized the whole venom effectively at a potency of 2.03 mg/mL (amount of venom completely neutralized by one milliliter of antivenom), equivalent to the normalized potency of 9.35 mg/g (amount of venom or toxin neutralized by one gram of antivenom protein). SSAV cross-neutralized the principal toxins i.e., SNTX and LNTX to different extents: the normalized potency values against SNTX and LNTX were 1.57 mg/g and 3.50 mg/g, respectively. All challenge doses (venom and toxins) were standardized at 5 LD_50_. The neutralization parameters i.e., median effective doses (ED_50_), median effective ratio (ER_50_), potency (P) and normalized potency (n-P) are shown in Table 4.

## 3. Discussion

C18 reverse-phase HPCL and gel electrophoresis revealed a common trend of venom profiles between *Hydrophis curtus* and related venomous marine snakes. This is consistent with the perceived “streamlining of venom” as an adaptive feature for predation in the aquatic environment, where fish is the main targeted prey. The venom chromatograms of *H. curtus* (current study) and its monophyletic cousin *H. schistosus* [12] both showed well resolved, less complex protein peaks in the following regions (based on an optimized venom-decomplexing protocol) [27]: 30–35 min, 60–75 min and 90–120 min, which corresponded to the elution of SNTX, LNTX and PLA_2_, respectively. The similar pattern of protein elution was reported for virtually all sea elapid venom profiles (using reverse-phase column), and the three toxin groups typically constitute the bulk of the venom proteins at >95% (by weight), although the individual protein subtypes and their individual expression could vary substantially at the inter- and intra-specific levels. Compared to *H. schistosus,* which has a more specialized diet (feeding mainly on tachysurus catfish), *H. curtus* is known to be generalistic, and a greater complexity of venom proteins is probably needed to subdue and digest different types of prey. This might be reflected in the more diverse protein types/subtypes and variable protein expression in *H. curtus* venom compared to *H. schistosus* profile at the subproteomic level.

On the whole, the venom proteomes of the congeneric *Hydrophis* sp. (*H. curtus*, *H. schistosus*, *H. platurus*, *H cyanocinctus*) and the paraphyletic *A. laevus* showed that 3FTX were invariably alpha-neurotoxins of short- and/or long-chain proteoforms (SNTX and LNTX). Alpha-neurotoxins are post-synaptic antagonists of post-junctional nicotinic acetylcholine receptors (nAChR); blockade of neurotransmission caused by these NTX can lead to neuromuscular paralysis, respiratory failure and death [28,29]. SNTX are generally more reversible than LNTX in receptor binding (inactivation of nAChR), but they are less well neutralized in vivo by commercial antivenoms, as shown in several toxin-specific neutralization studies [25,30,31]. The abundance of SNTX appears to be a limiting factor of antivenom efficacy (in neutralizing the venom toxicity), and this has an implication on antivenom production, i.e., that elapid antivenom efficacy should be improved by optimizing the formulation of toxin immunogen, taking into account the poorly neutralized neurotoxins. The Malaysian *H. curtus* venom (the object of the current study) has more SNTX than LTNX (SNTX:LNTX ratio of 10:1); this SNTX-predominating trend in the subproteome is observed in the congeneric *H. schistosus* (SNTX:LNTX ~4:1), *H. platura* (SNTX:LNTX ~2:1), *H. cyanocinctus* (SNTX:LNTX ~2:1), and the paraphyletic *A. laevus* which venom contained only SNTX [12,16,17,18]. On the other hand, the semi-marine *L. colubrina* (Bali) has more LNTX than SNTX (LNTX:SNTX ~3:1) in its venom [19], probably a feature related to its strict diet of eels. This could be an indicator for the evolutionary status of *L. colubrina* being basal to all other lineages, including the Australasian terrestrial elapids and the fully marine hydrophids nested within [21]. The three-finger neurotoxin profile is typically much more diverse in the terrestrial counterparts e.g., cobras [32], kraits [33] and coral snakes [34,35] but highly streamlined to SNTX in the hydrophids [12,17].

In the recently published venom proteome of *H. curtus* sourced from Weipa (Northern Australia), the alpha-NTX profile varied between two catches (January batch with body length >600 mm vs. June batch with body length <400 mm) [20]. In the January catch, that was categorized as adults (*n* = 10), the alpha-neurotoxins were predominantly LNTX (LNTX:SNTX ~3:1), whereas LNTX and SNTX were equally abundant in the June catch (subadults, *n* = 10) (LNTX:SNTX ~1:1). The profiles reported previously were varied from the current study in terms of the neurotoxin diversity and the ratio of SNTX to LNTX. The reverse-phase HPLC and the LCMS/MS data of *H. curtus* venoms also varied between the previous and the current studies. Notably, the first chromatographic peak (corresponding to SNTX elution) was remarkably much higher (with a larger AUC) followed by significantly lower peaks (10-fold smaller) of LNTX in the Malaysian sample, whereas in the Australian specimen, the main fractions containing LNTX (Fractions 2 and 3) showed comparable peak heights with its SNTX-containing Fraction 1 [20]. It was also noted that the neurotoxins (in particular LNTX) in the Australian *H. curtus* venom samples eluted across all chromatographic fractions (Fractions 1–11) in the previous study; this makes comparison on the elution pattern and protein abundance of NTX between different chromatograms rather challenging. The inconsistent resolution of NTX could be due to the different type of column materials used: the C18 column used in the present and most other venomic studies has a longer hydrocarbon chain bonded to the silica (as octadecylsilane); this packing material is inherently more hydrophobic and has a longer retention time for proteins compared to the C4 column. On reducing SDS-PAGE, the profiling of protein fractions (by molecular weights) from C18 reverse-phase HPLC (current study) was highly consistent with the protein identification by LCMS/MS for all fractions. Comparison of SDS-PAGE profiles for the venoms and corresponding HPLC fractions between the Malaysian and the Australian *H. curtus* was precluded, as the SDS-PAGE of Australian specimen was not available. Nonetheless, the proteomes reported thus far illustrate the remarkable variability of the highly evolved neurotoxins between the two geographical *H. curtus* populations.

Despite the differences in their 3FTX (neurotoxin) profile, PLA_2_ is consistently the most dominant proteins in the venoms of the Malaysian (>60%, current study) and the Australian *H. curtus* (55–67%) [20]. This phenomenon was also seen in *A. laevus* venom, in which PLA_2_ constituted >70% of the total venom proteins. The PLA_2_ dominance in these species is “balanced” with a lower 3FTX abundance, as shown in the Malaysian *H. curtus* (~26%, current study) and the Australian *A. laevus* (~25%) [18], although the Australian *H. curtus* venom contained a slightly higher abundance of 3FTX (30–40%) [20]. This venomic phenotype contrasts with the venom proteomes reported previously for the other core *Hydrophis* sea snakes (*H. cyanocinctus*, *H. schistosus* and *H. platurus*) [12,16,17], as well as the taxonomically-divergent *L. colubrina* [19], in which three-finger alpha-neurotoxins are dominant (50–81%). The unique subproteomic variability of *H. curtus* venom delineates a potential NTX-PLA_2_ dichotomy among the *Hydrophis* sea snakes, which should be further verified when more sea snake venom proteomes become available. The variable expression of neurotoxins and PLA_2_ presumably correlates with the venom neurotoxic and myotoxic activities, supporting the relationship between snake venom compositions and feeding adaptations in different niches. Clinically, the relatively lower alpha-neurotoxins abundance and higher PLA_2_ content imply that the pathophysiology of *H. curtus* envenoming possibly varies from other sea snake species that demonstrate a neurotoxin-predominating venom composition. Considering their high abundances, the PLA_2_ likely play a crucial role in the toxic activity of *H. curtus* venom. However, PLA_2_ is pharmacologically diverse [36], and the high number of PLA_2_ subtypes within the venom indicates that the PLA_2_ functionality is complex. In *H. curtus*, the predominating PLA_2_ are of acidic subtypes which were probably not as effective as sea snake basic PLA_2_ at penetrating the membrane phospholipids to cause cytotoxicity and myotoxicity [11,37]. The high acidic:basic PLA_2_ proportion in the Malaysian *H. curtus* venom proteome (4:1) is in line with proportions reported for the Australian *H. curtus* (3:1, 7:1) [20] and *A. laevus* (2:1) [18], but opposite to the Malaysian *H. schistosus* (1:4) [12] and the Balinese *L. colubrina,* whose venom virtually composed of basic PLA_2_ (acidic PLA_2_ was only present in a trace amount) [19]. Although it is generally well accepted that the basic PLA_2_ proteins contribute more substantially to venom lethality than acidic PLA_2_, the biological activities of the different PLA_2_ proteoforms (acidic or basic) can vary between snake species. The PLA_2_ activity may or may not be clinically important, as shown in several acidic or even basic PLA_2_ that are non-lethal from elapid venoms [19,25,31]. In studies of sea snake venom, the equivocal toxicity of PLA_2_ could be further elucidated with isolated toxin study (discussed below) to correlate with the clinical or epidemiological findings. In most sea snake envenoming, however, *H. curtus* bite has not been well identified and documented.

The diversity and the dominating proportion of *H. curtus* venom proteins in this study also differed from a previous work that studied cDNA libraries cloned from venom-gland mRNA of *H. curtus* (*Lapemis curtus*) sampled from Weipa, Australia [21]. The venom gene transcription appeared to bias toward 3FTX (43.4%) compared to PLA_2_ (9.8%), contrasting with the established venom proteome of this species (regardless of Weipa or Penang origin) in which PLA_2_ dominated (PLA_2_ > 3FTX, by total venom proteins). The incongruence between venom-gland transcriptome and venom proteome of snake has been well documented in several other species, such as the monocled cobra [38] and king cobra [39], indicating that there is significant post-transcription regulation and/or post-translational modification in the synthesis of snake venom proteins.

The cysteine-rich secretory protein (CRiSP) family contributes to a relatively high protein abundance in the Malaysian *H. curtus* venom. CRiSP was reported in the proteomes of virtually all sea snake venoms except for *H. cyanocinctus* [16], where its absence could be due to a lower detection sensitivity of mass spectrometry and instrument used previously. The abundance of CRiSP in the Malaysian *H. curtus* venom is consistent with that reported for the adult *H. curtus* (Weipa) and *A. laevus* (Broome). CRiSP exhibits diverse biological activities such as the inhibition of smooth muscle contraction, blockade of cyclic nucleotide-gated ion channel, and hypothermia in animals [40,41]. Clinically, the pathophysiological role of CRiSP in snakebite in humans is still unclear, and is worthy of further investigation. Characterization of the novel CRiSP from the Malaysian *H. curtus* venom is highly feasible in view of the abundance and purity of the protein (judged by its homogeneity on SDS-PAGE) eluted using the current HPLC protocol.

Other proteins present in the venom proteome were minor components (on average <1% each). The variety of these minor proteins, interestingly, is greater than in other sea elapids reported so far. The sea snake venom l-amino acid oxidase (LAAO) enzyme was detected proteomically for the second time in sea elapids, after the first report in the *H. schistosus* venom proteome [12]. This finding implies that LAAO could be a trace enzyme that exists in at least two species of the core *Hydrophis* genus, enriching our knowledgebase and demonstrating that sea snake venoms are not totally devoid of LAAO [42].

The detection of snake venom metalloproteinase (SVMP) in the Malaysian *H. curtus* venom is consistent with what had been observed in the venom proteomes of the Australian *H. curtus* and the Malaysian *H. schistosus* [12,20]. The contents of SVMP in the Malaysian *H. curtus* and *H. schistosus* were, however, markedly higher than the Australian *H. curtus*. As these were the four sea snake venom samples shown to contain SVMP thus far, it is uncertain whether the distribution of SVMP in sea snakes is geographically related. Besides, the Malaysian *H. curtus* venom contains phosphodiesterase, phospholipase B, Ophiophagus venom factor and waprin and this is the first report of the presence of these proteins in the venom proteomes of sea snakes, although weak phosphoesterase enzymatic activity had been reported previously from *Lapemis hardwickii* (*H. curtus*) venom [43]. The roles of these minor proteins in the venom remain to be further investigated.

In the in vivo toxicity test, *H. curtus* venom showed an approximately 2-fold higher LD_50_ compared to *H. schistosus* (LD_50_ = 0.07 µg/g) and *L. colubrina* (0.10 µg/g). In line with the current proteomic findings, the lower lethality of *H. curtus* venom is driven by its lower 3FTX content (alpha-neurotoxins, 26.33%) in contrast to the more lethal *H. schistosus* venom (alpha-neurotoxins, 70.5%; myotoxic PLA_2_, ~20%) and *L. colubrina* (alpha-neurotoxins, 66.14%) [12,19]. Considering the relative abundances of *H. curtus* SNTX (~22.89%) and LNTX (~3.44%), in one unit of LD_50_ of the whole venom (0.2 µg/g mouse), there are approximately 0.046 µg SNTX and 0.007 µg LNTX, which represent 46% and 2.9% of the LD_50_ of the neurotoxin subtypes, respectively. This indicates that SNTX and LNTX may work in synergism, contributing to the whole venom lethality, and antivenom treatment should seek to address effective neutralization of these specific principal toxins. On the other hand, the major PLA_2_ fractions in *H. curtus* venom lacked lethal activity; however, the potential myotoxic properties of the various PLA_2_ isoforms deserve further investigation in the future. On the whole, the sea snake venoms appeared to share similarities in their profiles and toxicity. In view of their relatively recent radiation and close phylogenetic relatedness, it is anticipated that substantial antigenicity is conserved among the different sea snake species. This immunological property of antigenicity sharing is medically important, as it would allow the use of SSAV (raised against the Malaysian *H. schistosus* venom) in cross-neutralizing the toxicity of different sea snake venoms. Earlier, in a nerve-muscle preparation, SSAV (1 unit/mL) added at t_90_ (time at which 90% inhibition of initial twitch height occurred) was able to reverse the inhibition of twitches (20–50%) produced by *H. curtus* venom [24]. In agreement with the in vitro findings, the present study further confirmed that SSAV was effective in vivo in cross-neutralizing the lethality of the Malaysian *H. curtus* venom. The normalized potency of SSAV against *H. curtus* (P = 9.35 mg/g) was in fact much higher than the previously reported potencies for *H. schistosus* (P = 2.21 mg/g) [25] and *Laticauda colubrina* (P = 5.01 mg/g) [19]. This could be due to the higher LD_50_ of *H. curtus* venom—at a challenge dose of 5 LD_50_, the neutralization could be interpreted as having more venom proteins being neutralized per unit antivenom. When examining the specific principal toxins, it was found that SSAV potency in neutralizing SNTX did not differ markedly among *H. schistosus*, *H. curtus* and *L. colubrina* (n-P = 1.2–1.5 mg/g) [19,25]. The neutralization of LNTX, however, varied to some extent (normalized potency was approximately 2–6 mg/g) among these species. The finding implies a conserved repertoire of SNTX epitopes among the species. The LNTX epitopes, on the other hand, seemed to be more variable, and the potency level of neutralization followed the homology of *H. schistosus* > *H. curtus* > *L. colubrina*. On toxin-specific neutralization, it is evident that SSAV exhibited better neutralization against LNTX than SNTX, indicating that SNTX is a limiting factor of antivenom efficacy. This phenomenon has also been reported in the neutralization of alpha-neurotoxins of several *Naja* cobra species (by cobra antivenoms) [25,30,31]. Further research is needed for the improvement of elapid antivenom formulation through strategies that aim to enhance the toxin immunogenicity and broaden the species coverage in the region [23].

The sharing of protein antigens between *H. curtus* and *H. schistosus* is further supported by the comparable immunoreactivity of SSAV toward both venoms. As the core hydrophids are a group of relatively young marine radiation of the elapids [3], the extent of amino acid substitution/divergence and protein neofunctionalization might be rather limited within the clade, hence the highly conserved protein antigenicity. The strong immunoreactivity of SSAV toward the venom of *N. scutatus* (Australian tiger snake), however, is inconsistent with the very different venom compositions (proteomes) between *N. scutatus* and *H. schistosus* [12,44]. The phenomenon could be partly explained by the fact that *N. scutatus* venom was included in the immunogen mix as a strategy to enhance the anti-titer level in SSAV production [44]. This observed strong immunoreactivity also supported the cross-neutralization of *N. scutatus* venom toxicity by SSAV previously [44]. On fraction-based ELISA, the strong binding of PLA_2_ and SNTX by SSAV indicates that the anti-titer against these proteins was high, presumably due to the higher abundance of these toxins in the venom (collectively > 90% of total proteins) used in immunization. Although SSAV neutralized LNTX more effectively than it neutralized SNTX, SSAV exhibited stronger immunoreactivity toward SNTX than to LNTX. This conflicting observation implies that the immunological binding activity of antivenom to toxins does not necessarily always correlate with the in vivo neutralization activity of antivenom. Hence, the neutralization capability and potency of antivenom against specific toxins should be tested in vivo in addition to in vitro immunological assays when studying antivenomics [45,46].

## 4. Conclusions

The venom proteome of Malaysian (Penang) *H. curtus* shows a minimalistic toxin arsenal that is dominated by PLA_2_ and 3FTX (composed of only short and long alpha-neurotoxins). The highly streamlined venom protein repertoire of the various *Hydrophis* sp. illustrates the case of molecular economy of toxins, which is an evolutionary solution convergently adopted by various sea snake taxa (including Laticaudinae) in response to the need for a fast moving, fish-based diet in the marine habitat. In comparison with the other marine/semi-marine species, *H. curtus* venom is unique, as there are more abundant and more diverse PLA_2_ than 3FTX (at a ratio of ~3:1) in the proteome. The major toxin distribution accounts for the higher LD_50_ of the venom, as its toxicity is mainly driven by the short alpha-neurotoxins (the diverse PLA_2_ were non-lethal). Subproteomic toxin variability within *H. curtus* of allopatric populations is apparent. The 3FTX of Malaysian *H. curtus* venom (Penang) comprised of mainly short neurotoxin, whereas the Australian specimens (Weipa) had more long-chain neurotoxins. The venom protein antigenicity was well conserved between *H. curtus* and the phylogenetically related *H. schistosus* (and *Notechis scutatus*) used in the production of SSAV, rendering the antivenom able to immunorecognize and in vivo cross-neutralize *H. curtus* venom effectively. The weak neutralization of short neurotoxin remains a limiting factor of antivenom efficacy in neutralization. Optimizing the immunogen formulation of alpha-neurotoxins may improve the efficacy of antivenom to neutralize NTX-predominating snake venoms.

## 5. Materials and Methods

### 5.1. Samples and Chemicals

*Hydrophis curtus* venom was a pooled sample from 10 adult snakes collected in the waters of Penang Island (Malaysia). The antivenom tested was Sea Snake Antivenom (SSAV) produced by CSL Ltd. (Melbourne, Australia, Seqirus Ltd. currently). The antivenom was in (liquid form) contained F(ab’)_2_ from horses hyperimmunized against the venoms of *Hydrophis schistosus* (beaked sea snake, obtained from the waters of Penang Island, Malaysia) and the Australian *Notechis scutatus* (common tiger snake). The protein concentration of the antivenom was 217.2 ± 3.0 mg/mL, determined in a previous study from the laboratory [25].

The chemicals and reagents used were primarily supplied by Sigma-Aldrich (Saint Louis, MO, USA) and were of analytical grade. The molecular weight marker Spectra™ Multicolor Broad Range Protein Ladder (10–260 kDa), trypsin (mass spectrometry grade), HPLC grade acetonitrile (ACN) and trifluoroacetic acid (TFA) were purchased from Thermo Scientific™ Pierce™ (Waltham, MA, USA). Millipore ZipTip^®^ C_18_ Pipette Tips and LiChrospher^®^ WP 300 RP-18 (5 µm) were supplied by Merck (Burlington, MA, USA).

### 5.2. Reverse-Phase High Performance Liquid Chromatography

Two milligrams of *H. curtus* venom were reconstituted in MilliQ ultrapure water and fractionated using a reverse-phase column (LiChrospher^®^ WP 300 C_18_ column, Merck Millipore, Burlington, MA, USA) via a high performance liquid chromatography system (Shimadzu LC-20AD, Shimadzu, Tokyo, Japan). The flow rate of mobile phase was set to 1 mL/min over a course of 180 min. Stepwise linear gradients of mobile phase composed of 0.1% TFA in water (Solvent A) and 0.1% TFA in 100% ACN (Solvent B) were used for protein elution as follow: 0–5% B for 10 min, 5–15% B for 20 min, 15–45% B for 120 min and 45–70% B over 20 min. The elution of protein was monitored at 215 nm. The protein fractions were collected manually, lyophilized and stored at −20 °C until use.

### 5.3. Sodium Dodecyl Sulphate-Polyacrylamide Gel Electrophoresis (SDS-PAGE)

The freeze-dried protein fractions collected from C18 reverse-phase HPLC were dissolved in MilliQ ultrapure water and further fractionated by electrophoresis on a 15% gel under reducing conditions. Coomassie Brilliant Blue stained the protein bands in each HPLC fractions on the gel. Spectra™ Multicolor Broad Range Protein Ladder (10 to 260 kDa) served as a standard for calibrating molecular weights of proteins.

### 5.4. In-Solution Protein Digestion with Trypsin

Fractionated proteins collected from the reverse-phase were reduced by DTT (Dithiothreitol), alkylated with IAA (iodoacetamide) and subjected to in-solution proteolytic digestion with trypsin according to the method reported previously [47]. Millipore ZipTip^®^ C_18_ Pipette Tips (Merck, Burlington, MA, USA) were used to clean up and desalt the trypsin-digested peptides for an enhanced performance of mass spectrometry analysis.

### 5.5. Nano-ESI-LCMS/MS of the Tryptic Digests and Label-Free LCMS/MS Protein Quantitation

The digested and desalted *H. curtus* venom peptides were analyzed using a nano-ESI LCMS/MS system (Agilent 1200 HPLC-Chip/MS Interface coupled with Agilent 6520 Accurate-Mass Q-TOF LC/MS, Agilent Technologies, Palo Alto, CA, USA). The peptides were loaded in a 300 Å C_18_, enrichment column (injection volume = 1 μL) followed by a 75 μm × 150 mm analytical column (Agilent N° G4240-62010). 0.1% formic acid in water (A) and 90% acetonitrile in water with 0.1% formic acid (B) were used for elution of the peptides at the following stepwise linear gradients: 3–50% B for 30 min, 50–95% B for 2 min, and 95% B for 5 min. The ion polarity was set to positive ionization mode. The temperature of the drying gas was 325 °C and the flow rate was 5 L/min. The fragmentor voltage and capillary voltage were set to 175 V and 1995 V, respectively. The ion spectra were acquired in an MS/MS mode where a MS scan range of 110–3000 *m*/*z* and a MS/MS scan range of 50–3000 *m*/*z* were used. Precursor charges were selected based on double- and triple-charged states but excluding the reference ions i.e., 922.0098 *m*/*z* (z = 1) and 121.0509 (z = 1). Data within the MH^+^ mass range of 600–4000 Da was obtained and processed with Agilent Spectrum Mill MS Proteomics Workbench software packages. A single modification was selected for the carbamidomethylation of cysteine. The peptide masses were searched against a non-redundant protein sequence database from NCBI (taxonomy: Serpentes, taxid: 8570) and an in-house transcriptomic database comprising elapid species of *Hydrophis curtus*, *Hydrophis schistosus, Ophiophagus hannah*, *Calliophis intestinalis*, *Bungarus caeruleus* and *Naja naja*. The identification of protein was validated using the following parameters: protein score >11, peptides score >6, scored peak intensity (SPI) >60%. The protein abundances were estimated through HPLC and ESI-LCMS/MS as previously described [47,48]. In brief, the relative abundance of an individual protein within a chromatographic fraction was estimated by its relative spectral intensity from LCMS/MS. The protein’s relative abundance in term of percentage of total venom proteins was then determined by multiplying its relative spectral intensity and its chromatographic peak area under the curve (AUC), as described previously.

### 5.6. Median Lethal Dose of Venom/Toxin and Efficacy of Neutralization by Antivenom

The venom proteins were injected intravenously into ICR mice via tail vein (20–25 g, *n* = 4 per dose, 4 doses). The mice were monitored and allowed free access to food and water ad libitum. The survival ratio of mice was recorded at 24 h. Neutralization assay was conducted as described previously [48]. A challenge dose of the venom proteins at 5 LD_50_ was mixed with various doses of Sea Snake Antivenom (SSAV) in normal saline and pre-incubated at 37 °C for 30 min, followed by tail vein injection (20–25 g, *n* = 4 per dose, 4 doses). The mice were allowed access to food and water ad libitum and the survival ratio was recorded at 24 h. Animal experiments were conducted according to the CIOMS guidelines [49]. Ethics clearance was given by the Institutional Animal Care and Use Committee, Faculty of Medicine, University of Malaya (Ethical approval code: 2016-190607/TCH/R/PHARM, Date of approval: 7 June 2016).

### 5.7. Immunological and Antigenic Profiling of H. curtus Venom Fractions

The immunoreactivity of Sea Snake Antivenom (SSAV) toward the HPLC protein fractions of *H. curtus* venom was examined using an indirect enzyme-linked immunosorbent assay (ELISA) as described previously [26]. The venoms of *H. schistosus* and *N. scutatus* were used as positive controls, while *Calloselasma rhodostoma* (Malayan pit viper) venom served as negative control. Immunoplate wells were each pre-coated with 10 ng proteins of the venom or venom protein fractions overnight at 4 °C. The plate was then flicked dry and rinsed three times with phosphate-buffered saline containing 0.5% Tween^®^20 (PBST). The protein concentrations of SSAV were prepared at 20 mg/mL, and 100 µL of SSAV in 1:900 PBST dilution was added to each pre-coated well, followed by 1 h incubation at room temperature. After washing the plate three times with PBST, 100 µL of appropriately diluted horseradish peroxidase-conjugated anti-horse-IgG (Jackson ImmunoResearch Inc., West Grove, PA, USA) in PBST (1:10000) was added to the well, followed by 1 h incubation at room temperature. The excess residues were washed away with PBST then. One hundred microliters of substrate solution (0.5 mg/mL o-phenylenediamine and 0.003% hydrogen peroxide in 0.1 M citrate-phosphate buffer, pH 5.0) was freshly prepared and added to each well. The enzymatic reaction took place in the dark for 30 min at room temperature. The reaction was then terminated by adding 50 µL of 12.5% sulphuric acid, and the absorbance at 490 nm was read against the blank using a multiplate reader (Molecular Devices-VersaMax^TM^, Sunnyvale, CA, USA). Values were means ± standard error mean (SEM) of triplicate experiments.

### 5.8. Statistical Analyses

The median lethal dose (LD_50_), median effective dose (ED_50_, the amount of reconstituted antivenom in µL that protects 50% of the animals tested), effective dose ratio (ER_50_, the amount of venom in mg neutralized per mL antivenom at which 50% of envenomed mice survived) and the 95% confidence intervals (C.I.) were calculated using the Probit analysis [50]. The potency of antivenom (P, defined as the amount of venom or toxin completely neutralized by a unit volume of antivenom, mg/mL), and the normalized potency values (n-P, defined as the amount of venom or toxin completely neutralized by a unit amount of antivenom, mg/g) were determined according to Tan et al. [51]. The statistical analysis package used was the BioStat analysis software (2008 v5.2.5, AnalystSoft Inc., Vancouver, BC, Canada).

## Figures and Tables

**Table 1:** Protein identification of Malaysian (Penang) *Hydrophis curtus* venom by ESI-LCMS/MS of C18 reverse-phase HPLC following in-solution tryptic digestion.

Fraction	Distinct Peptides	MS/MS Search Score	Species (as Per Annotation in Database)	Database Accession ^a^	Protein Name (as Per Annotation in Database)	Relative Abundance (%) ^b^
1	2	28.12	*Hydrophis curtus*	P68416	Short neurotoxin 1	22.89
2	3	62.47	*Hydrophis curtus*	A3FM53	Long neurotoxin 2	0.49
3	5	114.27	*Hydrophis curtus*	Q8UW29	Long neurotoxin 1	1.10
	4	84.14	*Hydrophis curtus*	A3FM53	Long neurotoxin 2	1.32
	2	40.42	*Ophiophagus hannah*	Q53B58	Long neurotoxin OH-55	0.33
	1	21.62	*Ophiophagus hannah*	Q53B57	Long neurotoxin OH-56	0.21
4	5	92.36	*Hydrophis curtus*	Q8UW31	Acidic phospholipase A_2_ 57	0.08
	2	46.47	*Enhydrina schistosa*	Unigene22561_ESM	Acidic phospholipase A_2_ 57	0.34
	2	43.43	*Hydrophis curtus*	CL4079.Contig1_HCM	Basic phospholipase A_2_ 73	0.07
	2	35.82	*Calliophis intestinalis*	CL2932.Contig3_CIM	Acidic phospholipase A_2_ 57	0.16
	3	66.02	*Enhydrina schistosa*	P00610	Basic phospholipase A_2_	0.45
	2	50.61	*Hydrophis curtus*	Q8UW30	Basic phospholipase A_2_ 73	0.32
	1	20.76	*Hydrophis curtus*	Unigene14087_HCM	Waprin-Rha1	0.11
5	4	85.11	*Enhydrina schistosa*	P00610	Basic phospholipase A_2_	2.90
	3	64.58	*Hydrophis curtus*	Q8UW08	Basic phospholipase A_2_	1.68
	3	60.39	*Hydrophis curtus*	Q8UW30	Basic phospholipase A_2_ 73	2.78
	2	46.67	*Enhydrina schistosa*	Unigene22561_ESM	Acidic phospholipase A_2_ 57	1.80
	2	45.14	*Naja Naja*	CL339.Contig1_NNSL	Basic phospholipase A_2_	2.85
	3	47.42	*Hydrophis curtus*	Q8UW31	Acidic phospholipase A_2_ 57	0.48
	2	31.85	*Bungurus caeruleus*	Unigene17389_BCSL	Acidic phospholipase A_2_ 57	0.05
	2	24.1	*Calliophis intestinalis*	CL2932.Contig3_CIM	Acidic phospholipase A_2_ 57	0.15
6	7	131.08	*Hydrophis curtus*	Q8UW31	Acidic phospholipase A_2_ 57	28.11
	2	46.96	*Hydrophis curtus*	Q8UW08	Basic phospholipase A_2_	0.98
	2	42.22	*Enhydrina schistosa*	P00610	Basic phospholipase A_2_	0.80
	2	41.03	*Bungarus caeruleus*	Unigene17389_BCSL	Acidic phospholipase A_2_ 57	15.26
	2	38.92	*Hydrophis curtus*	Q8UW30	basic phospholipase A_2_ 73	0.91
	2	31.08	*Denisonia devisi*	R4G2S8	PLA-2-Den-2	1.82
7	13	242.58	*Hydrophis curtus*	CL2848.Contig2_HCM	Extracellular matrix protein 1	0.16
8	8	143.01	*Hydrophis curtus*	CL2323.Contig2_HCM	Lysozyme C, milk isozyme-like	0.77
9	8	148.33	*Hydrophis curtus*	Q8UW11	Cysteine-rich venom protein 2	3.28
	6	111.6	*Enhydrina schistosa*	CL131.Contig1_ESM	Cysteine-rich secretory protein	3.42
	3	51.38	*Hydrophis curtus*	Q8UW25	Cysteine-rich venom protein 1	1.49
10	23	394.95	*Ophiophagus hannah*	P81383	L-amino-acid oxidase	0.18
	20	351.67	*Hydrophis curtus*	Unigene7803_HCM	Transferrin	0.15
	12	197.37	*Ophiophagus hannah*	I2C090	Ophiophagus venom factor	0.06
	8	139.98	*Ophiophagus hannah*	CL304.Contig1_OHM	OVF precursor protein	0.05
	9	144.94	*Hydrophis curtus*	CL4561.Contig1_HCM	Glutathione peroxidase 3	0.07
	8	138.68	*Enhydrina schistosa*	CL1665.Contig1_ESM	Sulfhydryl oxidase 1-like	0.07
	7	124.2	*Hydrophis curtus*	Q8UW11	Cysteine-rich venom protein 2	0.81
	7	122.36	*Hydrophis curtus*	CL4690.Contig9_HCM	Phospholipase A2 inhibitor	0.20
	6	89.52	*Ovophis okinavensis*	U3TDL2	glutaminyl_cyclase	0.02
	4	69.73	*Hydrophis curtus*	Unigene19328_HCM	Phospholipase A2 inhibitor beta	0.25
	4	54.96	*Hydrophis curtus*	CL2048.Contig1_HCM	Multiple inositol polyphosphate phosphatase 1	0.05
	3	46.73	*Hydrophis curtus*	CL1278.Contig2_HCM	Phospholipase B	0.02
	3	45.91	*Enhydrina schistosa*	CL560.Contig2_ESM	Carinatease-1	0.09
	2	37.92	*Hydrophis curtus*	CL4690.Contig1_HCM	Scutatease-1	0.22
	2	35.54	*Hydrophis curtus*	Unigene390_HCM	Zinc metalloproteinase-disintegrin-like NaMP	0.05
	2	36.44	*Enhydrina schistosa*	CL79.Contig2_ESM	ADP-ribosyl cyclase 1	0.01
	2	34.29	*Enhydrina schistosa*	CL98.Contig1_ESM	Lysosomal Pro-X carboxypeptidase-like	0.04
	2	32.92	*Hydrophis curtus*	Unigene20804_HCM	Phosphodiesterase	0.04
	2	32.47	*Hydrophis curtus*	CL1263.Contig2_HCM	Golgi apparatus protein 1	0.02
	2	30.78	*Hydrophis curtus*	Unigene23143_HCM	N-acetylglucosamine-6-sulfatase	0.02
	2	29.28	*Ophiophagus hannah*	CL2083.Contig1_OHM	OVF precursor protein	0.03

^a^ Protein codes with suffix “_HCM”, “_ESM”, “_OHM”, “_CIM”, “_BCSL” and “_NNSL” were derived from the in-house database containing RNAseq specific for the following: HCM: Malaysian *Hydrophis curtus*; ESM: Malaysian *Hydrophis schistosus*; OHM: Malaysian *Ophiophagus hannah*; CIM: Malaysian *Calliophis intestinalis*; BCSL: Sri Lankan *Bungarus caeruleus*; NNSL: Sri Lankan *Naja naja*. ^b^ Protein abundance was interpreted as the percentage of total venom proteins. Cysteine residues determined in MS/MS analysis are carbamidomethylated. Protein identifications were validated with the following filters: protein score >11, peptides score >6 and scored peak intensity (SPI) >60%. The relative abundance of an individual protein was estimated based on its relative spectral intensity within an HPLC fraction and the chromatographic peak area under the curve (AUC).

**Table 2:** Overview of Malaysian (Penang) *Hydrophis curtus* venom proteome by protein families and subtypes with relative abundances (%).

Protein Family/Protein Subtype	Fraction	Accession Code ^a^	Relative Abundance (%) ^b^
**Phospholipases A_2_**			**62.00**
**Acidic PLA_2_**			**48.26**
Acidic phospholipase A_2_ 57	4,5,6	Q8UW31	28.68
PLA-_2_-Den-2	6	R4G2S8	1.82
Acidic phospholipase A_2_ 57	5,6	Unigene17389_BCSL	15.31
Acidic phospholipase A_2_ 57	4,5	Unigene22561_ESM	2.15
Acidic phospholipase A_2_ 57	4,5	CL2932.Contig3_CIM	0.31
**Basic PLA_2_**			**13.74**
Basic phospholipase A_2_	4,5,6	P00610	4.15
Basic phospholipase A_2_	5,6	Q8UW08	2.66
Basic phospholipase A_2_ 73	4,5,6	Q8UW30	4.01
Basic phospholipase A_2_	5	CL339.Contig1_NNSL	2.85
Basic phospholipase A_2_ 73	4	CL4079.Contig1_HCM	0.07
**Three-finger toxins**			**26.33**
**Short Neurotoxin**			**22.89**
Short neurotoxin 1	1	P68416	22.89
**Long Neurotoxin**			**3.44**
Long neurotoxin 2	2,3	A3FM53	1.81
Long neurotoxin OH-56	3	Q53B57	0.21
Long neurotoxin OH-55	3	Q53B58	0.33
Long neurotoxin 1	3	Q8UW29	1.10
**Cysteine-rich secretory protein**			**9.00**
Cysteine-rich venom protein 2	9,10	Q8UW11	4.09
Cysteine-rich venom protein 1	9	Q8UW25	1.49
Cysteine-rich secretory protein	9	CL131.Contig1_ESM	3.42
**Phospholipase A_2_ inhibitors**			**0.45**
Phospholipase A_2_ inhibitor beta	10	Unigene19328_HCM	0.25
Phospholipase A_2_ inhibitor	10	CL4690.Contig9_HCM	0.20
**Snake venom metalloproteinases**			**0.36**
Zinc metalloproteinase-disintegrin-like NaMP	10	Unigene390_HCM	0.05
Scutatease-1	10	CL4690.Contig1_HCM	0.22
Carinatease-1	10	CL560.Contig2_ESM	0.09
**L-amino acid oxidase**			**0.18**
L-amino-acid oxidase	10	P81383	0.18
**Ophiophagus venom factor**			**0.14**
Ophiophagus venom factor	10	I2C090	0.06
OVF precursor protein	10	CL304.Contig1_OHM	0.05
OVF precursor protein	10	CL2083.Contig1_OHM	0.03
**Waprin**			**0.11**
Waprin-Rha1	4	Unigene14087_HCM	0.11
**Phosphodiesterase**			**0.04**
Phosphodiesterase	10	Unigene20804_HCM	0.04
**Phospholipase B**			**0.02**
Phospholipase B	10	CL1278.Contig2_HCM	0.02
**Non-toxin**			**1.38**
Extracellular matrix protein 1	7	CL2848.Contig2_HCM	0.16
Lysozyme C, milk isozyme-like	8	CL2323.Contig2_HCM	0.77
Transferrin	10	Unigene7803_HCM	0.15
Glutathione peroxidase 3	10	CL4561.Contig1_HCM	0.07
Sulfhydryl oxidase 1-like	10	CL1665.Contig1_ESM	0.07
Glutaminyl_cyclase	10	U3TDL2	0.02
Multiple inositol polyphosphate phosphatase 1	10	CL2048.Contig1_HCM	0.05
ADP-ribosyl cyclase 1	10	CL79.Contig2_ESM	0.01
Lysosomal Pro-X carboxypeptidase-like	10	CL98.Contig1_ESM	0.04
Golgi apparatus protein 1	10	CL1263.Contig2_HCM	0.02
N-acetylglucosamine-6-sulfatase	10	Unigene23143_HCM	0.02

^a^ Protein codes with suffix “_HCM”, “_ESM”, “_OHM”, “_CIM”, “_BCSL” and “_NNSL” were derived from the in-house database containing RNAseq specific for the following: HCM: Malaysian *Hydrophis curtus*; ESM: Malaysian *Hydrophis schistosus*; OHM: Malaysian *Ophiophagus hannah*; CIM: Malaysian *Calliophis intestinalis*; BCSL: Sri Lankan *Bungarus caeruleus*; NNSL: Sri Lankan *Naja naja*. ^b^ Protein abundance was interpreted as the percentage of total venom proteins.

**Table 3:** Venom proteomic profiles of sea elapids (sea snakes and sea krait). Values indicate the relative abundances of the proteins (by total venom protein).

Species	*H. curtus* (Penang) *n* = 10 Adults Dry Weight Per Milking: ~1–8 mg	*H. curtus* (January, Weipa) *n* = 11 Adults	*H. curtus* (June, Weipa) *n* = 10 Subadults	*H. schistosus* (Penang) *n* = 10 Adults	*L. colubrina* (Bali) *n* = Several	*Aipysurus laevus* (Broome) *n* = 4	*H. platura* (Costa Rica) *n* = 84	*H. cyanocinctus* (Hara) *n* = Several
**i.v. LD_50_ (µg/g mouse)**	0.20 (0.18–0.24)	NA	NA	0.07 (0.05–0.09)	0.10 (0.08–0.12)	0.15 (0.08–0.25)	0.23 (note: 0.13 by i.p. route)	0.132
**Reference**	Current study	Neale et al. (2017) [20]		Tan et al. (2015b) [12]	Tan et al. (2017) [19]	Laustsen et al. (2015) [18]	Lomonte et al. (2014) [17]	Calvete et al. (2012) [16]
**Methods**	C18 rpHPLC, in-solution tryptic digestion, LCMS/MS	C4 rpHPLC, in-solution tryptic digestion, LCMS/MS	C4 rpHPLC, in-solution tryptic digestion, LCMS/MS	C18 rpHPLC, in-gel tryptic digestion, MALDI TOF/TOF	C18 rpHPLC, in-solution tryptic digestion, LCMS/MS	C18 rpHPLC, in-gel tryptic digestion, MALDI TOF/TOF	C18 rpHPLC, in-gel tryptic digestion, LCMS/MS	C18 rpHPLC, N-terminal sequencing, in-gel tryptic digestion, CID-MS/MS
**3FTX**	26.33	30.44	40.43	70.5	66.14	25.3	~49.9	81.1
*SNTX*	*22.89*	*8.33*	*20.76*	*55.8*	*16.94*	*25.3*	*~36*	*51.7*
*LNTX*	*3.44*	*22.11*	*19.67*	*14.7*	*48.9*	*-*	*~14*	*29.4*
*CTX*	*-*	*-*	*-*	*-*	*0.3*		*-*	*-*
**PLA_2_**	62.00	66.7	54.50	27.5	33.3	71.2	32.9	18.9
*Acidic*	*48.26*	*57.93*	*41.21*	*6.1*	*0.04*	*46.4*	*NA*	*NA*
*Basic*	*13.74*	*8.76*	*13.28*	*21.4*	*33.26*	*24.8*	*NA*	*NA*
*Neutral*	*-*	*0.01*	*0.007*	*-*	*-*	*-*	*NA*	*NA*
**CRISP**	9.00	2.53	4.95	1.3	0.05	2.5	9.1	-
**LAAO**	0.18	-	-	0.2	-	-	-	-
**SVMP**	0.36	0.004	0.01	0.5	-	-	~0.9	-
**NUC**	-	-	-	-	-	-	~0.8	-
**CTL**	-	0.09	0.003	-	-	-	-	-
**PDE**	0.04	-	-	-	-	-	-	-
**PLB**	0.02	-	-	-	-	-	-	-
**PLA_2_ inhibitor**	0.45	0.01	-	-	-	-	-	-
**OVF**	0.14	-	-	-	-	-	-	-
**Waprin**	0.11	-	-	-	-	-	-	-
**Non-toxins/cellular proteins**	1.38	0.231	0.12	-	-	0.2	5.0	-
**Unknown**	-	-	-	-	0.57	0.8	1.4	-

Abbreviations: LD_50_: median lethal dose; i.v.: intravenous; i.p.: intraperitoneal; rpHPLC: reverse-phase high performance liquid chromatography; LCMS/MS: Liquid chromatography-tandem mass spectrometry; MALDI TOF/TOF: Matrix assisted laser desorption/ionization-time of flight tandem mass spectrometry; CID-MS/MS: Collision-induced dissociation-tandem mass spectrometry; 3FTX: three-finger toxin; SNTX: Short alpha-neurotoxin; LNTX: Long alpha-neurotoxin; CTX: Cytotoxin; PLA_2_: Phospholipase A_2_; CRISP: Cysteine-rich secretory protein; LAAO: l-amino acid oxidase; SVMP: Snake venom metalloproteinase; NUC: Nucleotidase; CTL: C-type lectin; PDE: Phosphodiesterase; PLB: Phospholipase B; OVF: Ophiophagus venom factor. NA: Not available.

**Table 4:** Efficacy and potency of Seqirus Sea Snake Antivenom (SSAV) in cross-neutralizing the lethal effect of *Hydrophis curtus* venom and toxin fractions.

Venom/Fraction	Challenge Dose	i.v. LD_50_ (µg/g) ^a^	ED_50_ (µL) ^b^	ER_50_ (mg/mL) ^c^	Potency, P (mg/mL) ^d^	SSAV Protein Concentration (mg/mL)	Normalized P, n-P (mg/g) ^e^
Venom	5	0.20 (0.18–0.24)	9.87 (7.98–12.21)	2.53 (2.28–3.04)	2.03 (1.83–2.43)	217.2 ± 3.0	9.35
F1_SNTx	5	0.10 (0.08–0.12)	25.82 (22.38–29.79)	0.41 (0.34–0.51)	0.34 (0.27–0.41)	217.2 ± 3.0	1.57
F3_LNTx	5	0.24 (0.21–0.28)	27.90 (25.36–30.70)	0.95 (0.83–1.10)	0.76 (0.66–0.88)	217.2 ± 3.0	3.50
F5	-	>1	-	-	-	-	-
F6	-	>1	-	-	-	-	-
F9	-	>1	-	-	-	-	-

LD_50_: Median lethal dose; ED_50_: Median effective dose; ER_50_: Median effective ratio; ^a^ Median lethal dose was defined as the dose of venom (µg/mL) at which 50% of mice died. ^b^ Median effective dose was defined as the dose of antivenom (µL) at which 50% of mice survived. ^c^ Median effective ratio was defined as the ratio of venom (mg) to the volume does of antivenom (mL) at which 50% of mice survived. ^d^ Potency, P, the neutralization potency of antivenom (mg/mL) was defined as the amount of venom (mg) that was completely neutralized by one mL of antivenom. ^e^ Normalized P, n-P was defined as the neutralization potency of the antivenom in mg venom/g antivenom.

## Supplementary Materials

The following is available online at http://www.mdpi.com/2072-6651/11/1/3/s1, Table S1: Mass spectrometry results of venom from *Hydrophis curtus* (Penang, Malaysia).

## References

[1] Slowinski J.B., Keogh J.S. (2000). Phylogenetic relationships of elapid snakes based on cytochrome b mtDNA sequences. Mol. Phylogenet. Evol..

[2] Sanders K.L., Lee M.S., Leys R., Foster R., Keogh J.S. (2008). Molecular phylogeny and divergence dates for Australasian elapids and sea snakes (hydrophiinae): Evidence from seven genes for rapid evolutionary radiations. J. Evol. Biol..

[3] Sanders K.L., Lee M.S., Mumpuni, Bertozzi T., Rasmussen A.R. (2013). Multilocus phylogeny and recent rapid radiation of the viviparous sea snakes (Elapidae: Hydrophiinae). Mol. Phylogenet. Evol..

[4] Reid H.A. (1975). Epidemiology of sea-snake bites. J. Trop. Med. Hyg..

[5] Reid H.A., Lim K.J. (1957). Sea-snake bite; a survey of fishing villages in northwest Malaya. Br. Med. J..

[6] Kularatne S.A., Hettiarachchi R., Dalpathadu J., Mendis A.S., Appuhamy P.D., Zoysa H.D., Maduwage K., Weerasinghe V.S., de Silva A. (2014). *Enhydrina schistosa* (Elapidae: Hydrophiinae) the most dangerous sea snake in Sri Lanka: Three case studies of severe envenoming. Toxicon.

[7] Vithanage K.K., Thirumavalavan K. (2012). A case of a sea snake bite resulting in fatal envenoming. Ceylon Med. J..

[8] Cao N., Tao N.T., Moore A., Montoya A., Rasmussen A.R., Broad K., Voris H.K., Takacs Z. (2014). Sea Snake Harvest in the Gulf of Thailand. Conserv. Biol..

[9] WHO (2016). Guidelines for the Management of Snake Bites.

[10] Reid H.A., Lee C.-Y. (1979). Symptomatology, Pathology and Treatment of the Bites of Sea Snakes. Snake Venoms.

[11] Gopalakrishnakone P., Ponraj D., Thwinn M.M. (1997). Myotoxic Phospholipases from Snake Venoms: General Myoglobinuric and Local Myonecrotic Toxins.

[12] Tan C.H., Tan K.Y., Lim S.E., Tan N.H. (2015). Venomics of the beaked sea snake, *Hydrophis schistosus*: A minimalist toxin arsenal and its cross-neutralization by heterologous antivenoms. J. Proteom..

[13] Brook G.A., Torres L.F., Gopalakrishnakone P., Duchen L.W. (1987). Effects of phospholipase of *Enhydrina schistosa* venom on nerve, motor end-plate and muscle of the mouse. Q. J. Exp. Physiol..

[14] Mackessy S.P., Tu A.T., Tu A.T. (1993). Biology of the sea snakes and biochemistry of their venoms. Toxin-Related Diseases: Poisons Originating from Plants, Animals and Spoilage.

[15] Fry B.G., Wuster W., Ryan Ramjan S.F., Jackson T., Martelli P., Kini R.M. (2003). Analysis of Colubroidea snake venoms by liquid chromatography with mass spectrometry: Evolutionary and toxinological implications. Rapid Commun. Mass Spectrom..

[16] Calvete J.J., Ghezellou P., Paiva O., Matainaho T., Ghassempour A., Goudarzi H., Kraus F., Sanz L., Williams D.J. (2012). Snake venomics of two poorly known Hydrophiinae: Comparative proteomics of the venoms of terrestrial *Toxicocalamus longissimus* and marine *Hydrophis cyanocinctus*. J. Proteom..

[17] Lomonte B., Pla D., Sasa M., Tsai W.C., Solorzano A., Urena-Diaz J.M., Fernandez-Montes M.L., Mora-Obando D., Sanz L., Gutierrez J.M. (2014). Two color morphs of the pelagic yellow-bellied sea snake, *Pelamis platura*, from different locations of Costa Rica: Snake venomics, toxicity, and neutralization by antivenom. J. Proteom..

[18] Laustsen A.H., Gutierrez J.M., Rasmussen A.R., Engmark M., Gravlund P., Sanders K.L., Lohse B., Lomonte B. (2015). Danger in the reef: Proteome, toxicity, and neutralization of the venom of the olive sea snake, *Aipysurus laevis*. Toxicon.

[19] Tan C.H., Wong K.Y., Tan K.Y., Tan N.H. (2017). Venom proteome of the yellow-lipped sea krait, *Laticauda colubrina* from Bali: Insights into subvenomic diversity, venom antigenicity and cross-neutralization by antivenom. J. Proteom..

[20] Neale V., Sotillo J., Seymour J.E., Wilson D. (2017). The venom of the spine-bellied sea snake (*Hydrophis curtus*): Proteome, Toxin diversity and intraspecific variation. Int. J. Mol. Sci..

[21] Pahari S., Bickford D., Fry B.G., Kini R.M. (2007). Expression pattern of three-finger toxin and phospholipase A_2_ genes in the venom glands of two sea snakes, *Lapemis curtus* and *Acalyptophis peronii*: Comparison of evolution of these toxins in land snakes, sea kraits and sea snakes. BMC Evol. Biol..

[22] Takasaki C., Kimura S., Kokubun Y., Tamiya N. (1988). Isolation, properties and amino acid sequences of a phospholipase A_2_ and its homologue without activity from the venom of a sea snake, *Laticauda colubrina*, from the Solomon Islands. Biochem. J..

[23] Ratanabanangkoon K., Tan K.Y., Eursakun S., Tan C.H., Simsiriwong P., Pamornsakda T., Wiriyarat W., Klinpayom C., Tan N.H. (2016). A simple and novel strategy for the production of a pan-specific antiserum against Elapid snakes of Asia. PLoS Negl. Trop. Dis..

[24] Chetty N., Du A., Hodgson W.C., Winkel K., Fry B.G. (2004). The in vitro neuromuscular activity of Indo-Pacific sea-snake venoms: Efficacy of two commercially available antivenoms. Toxicon.

[25] Tan K.Y., Tan C.H., Fung S.Y., Tan N.H. (2016). neutralization of the principal toxins from the venoms of Thai *Naja kaouthia* and Malaysian *Hydrophis schistosus*: Insights into toxin-specific neutralization by two different antivenoms. Toxins.

[26] Tan C.H., Tan N.H., Tan K.Y., Kwong K.O. (2015). Antivenom cross-neutralization of the venoms of *Hydrophis schistosus* and *Hydrophis curtus*, two common sea snakes in Malaysian waters. Toxins.

[27] Tan C.H., Tan K.Y., Tan N.H. (2019). A Protein Decomplexation strategy in snake venom proteomics. Methods Mol. Biol..

[28] Tan K.Y., Tan C.H., Sim S.M., Fung S.Y., Tan N.H. (2016). Geographical venom variations of the Southeast Asian monocled cobra (*Naja kaouthia*): Venom-induced neuromuscular depression and antivenom neutralization. Comp. Biochem. Physiol. C Toxicol. Pharmacol..

[29] Barber C.M., Isbister G.K., Hodgson W.C. (2013). Alpha neurotoxins. Toxicon.

[30] Tan N.H., Wong K.Y., Tan C.H. (2017). Venomics of *Naja sputatrix*, the Javan spitting cobra: A short neurotoxin-driven venom needing improved antivenom neutralization. J. Proteom..

[31] Wong K.Y., Tan C.H., Tan N.H. (2016). Venom and Purified Toxins of the Spectacled Cobra (*Naja naja*) from Pakistan: Insights into Toxicity and Antivenom Neutralization. Am. J. Trop. Med. Hyg..

[32] Wong K.Y., Tan C.H., Tan K.Y., Quraishi N.H., Tan N.H. (2018). Elucidating the biogeographical variation of the venom of *Naja naja* (spectacled cobra) from Pakistan through a venom-decomplexing proteomic study. J. Proteom..

[33] Oh A.M.F., Tan C.H., Ariaranee G.C., Quraishi N., Tan N.H. (2017). Venomics of *Bungarus caeruleus* (Indian krait): Comparable venom profiles, variable immunoreactivities among specimens from Sri Lanka, India and Pakistan. J. Proteom..

[34] Tan K.Y., Liew J.L., Tan N.H., Quah E.S.H., Ismail A.K., Tan C.H. (2018). Unlocking the secrets of banded coral snake (*Calliophis intestinalis*, Malaysia): A venom with proteome novelty, low toxicity and distinct antigenicity. J. Proteom..

[35] Lomonte B., Rey-Suarez P., Fernandez J., Sasa M., Pla D., Vargas N., Benard-Valle M., Sanz L., Correa-Netto C., Nunez V. (2016). Venoms of *Micrurus* coral snakes: Evolutionary trends in compositional patterns emerging from proteomic analyses. Toxicon.

[36] Doley R., Zhou X., Kini R.M. (2009). Snake Venom Phospholipase A_2_ Enzymes.

[37] Geh S.L., Toh H.T. (1978). Ultrastructural changes in skeletal muscle caused by a phospholipase A2 fraction isolated from the venom of a sea snake, *Enhydrina schistosa*. Toxicon.

[38] Tan K.Y., Tan C.H., Chanhome L., Tan N.H. (2017). Comparative venom gland transcriptomics of *Naja kaouthia* (monocled cobra) from Malaysia and Thailand: Elucidating geographical venom variation and insights into sequence novelty. PeerJ.

[39] Tan C.H., Tan K.Y., Fung S.Y., Tan N.H. (2015). Venom-gland transcriptome and venom proteome of the Malaysian king cobra (*Ophiophagus hannah*). BMC Genom..

[40] Yamazaki Y., Morita T. (2004). Structure and function of snake venom cysteine-rich secretory proteins. Toxicon.

[41] Heyborne W.H., Mackessy S.P., Mackessy S.P. (2009). Cysteine-rich secretory proteins in reptile venoms. Handbook of Venoms and Toxins of Reptiles.

[42] Tan C.H., Tan N.H., Gopalakrishnakone P., Inagaki H., Mukherjee A.K., Rahmy T.R., Vogel C.-W. (2015). Toxinology of Snake Venoms: The Malaysian Context. Snake Venoms.

[43] Tan N.H., Ponnudurai G. (1991). A comparative study of the biological properties of some sea snake venoms. Comp. Biochem. Physiol. B.

[44] Tan C.H., Tan K.Y., Tan N.H. (2016). Revisiting *Notechis scutatus* venom: On shotgun proteomics and neutralization by the "bivalent" Sea Snake Antivenom. J. Proteom..

[45] Gutiérrez J.M., Lomonte B., León G., Alape-Girón A., Flores-Díaz M., Sanz L., Angulo Y., Calvete J.J. (2009). Snake venomics and antivenomics: Proteomic tools in the design and control of antivenoms for the treatment of snakebite envenoming. J. Proteom..

[46] Faisal T., Tan K.Y., Sim S.M., Quraishi N., Tan N.H., Tan C.H. (2018). Proteomics, functional characterization and antivenom neutralization of the venom of Pakistani Russell’s viper (*Daboia russelii*) from the wild. J. Proteom..

[47] Tan C.H., Tan K.Y., Yap M.K., Tan N.H. (2017). Venomics of *Tropidolaemus wagleri*, the sexually dimorphic temple pit viper: Unveiling a deeply conserved atypical toxin arsenal. Sci. Rep..

[48] Tan C.H., Liew J.L., Tan K.Y., Tan N.H. (2016). Assessing SABU (Serum Anti Bisa Ular), the sole Indonesian antivenom: A proteomic analysis and neutralization efficacy study. Sci. Rep..

[49] Howard-Jones N. (1985). A CIOMS ethical code for animal experimentation. WHO Chron.

[50] Finney D.J. (1952). Probit Analysis.

[51] Tan K.Y., Tan N.H., Tan C.H. (2018). Venom proteomics and antivenom neutralization for the Chinese eastern Russell’s viper, *Daboia siamensis* from Guangxi and Taiwan. Sci. Rep..

